# Applications of nanoparticels in molecular and cellular biology and cancer research

**DOI:** 10.1186/1753-6561-5-S8-P58

**Published:** 2011-11-22

**Authors:** Katya Simeonova, Ganka Milanova

**Affiliations:** 1 Institute of Mechanics, Bulgarian Academy of Sciences, 1113 Sofia, Bulgaria; 2 University of Architecture, Civil Engineering and Geodesy, 1000 Sofia, Bulgaria

## Background

Discovering of carbon nanotubes (CNTs), by S. Iijima in 1991, [1] was a revolution in nanoscience (nanomaterials) and nanotechnology. Moreover these nanoscale materials possess perfectly physico-mechanicanical, electronic, optical, properties. They find applications in technique, engineering, electronics, optoelectronis, space and environments, [2]. Recently has been established that nanomaterials, play an important role in molecular and cellular biology and medicine. The aim of the work presented could be formulated as follows: to discuss some basic articles, devoted to applications of nanoparticles, nanotechnology based on gold nanoparticles for cancer research.

## Materials and methods

Synthesis methods for nanoparticles (nanoshells, nanorods, nanocrystals) have been analyzed. Application of gold nanoparticels for detection and therapy of cancer has been given too, [3]. Nanotechnology has been determined as an interdisciplinary science combining physics, mechanics, chemistry, materials science, engineering, biology becames a very good potential in many different fields of technique, for cancer therapy, in molecular and cellular biology.

## Methods for synthesis of nanoparticles

Some methods for synthesis of nanoparticles: by controlled different reducing agent; a two- phase method using other reductants; biocompatible bock polymers. Deposition process (DP), has been applied for synthesis also. It has been established that rods wires, multi-concentric shells, hollow tubes, capsules, monocrystas etc. possess exceptional optical and electronic properties. In [4], the Drude method (model) for description of optical properties is:(1)(2)

Here: *ε*′ and *ε*″ ; *ω* = 2*πc*/*λ*; *λ, c, γ* have been given in [5]. A correlation, available is:(3)

Here *γ*, *γ_bulk_*, *ν_F_*, *r_off_* are given in [5]. Both important characteristics for description of gold nanoparticles, absorption efficiency and scattering efficiency have been analyzed too. It must be pointed out as well, that these nanoparticles could employed in the many medical applications.

## Results

**Figure 1:**
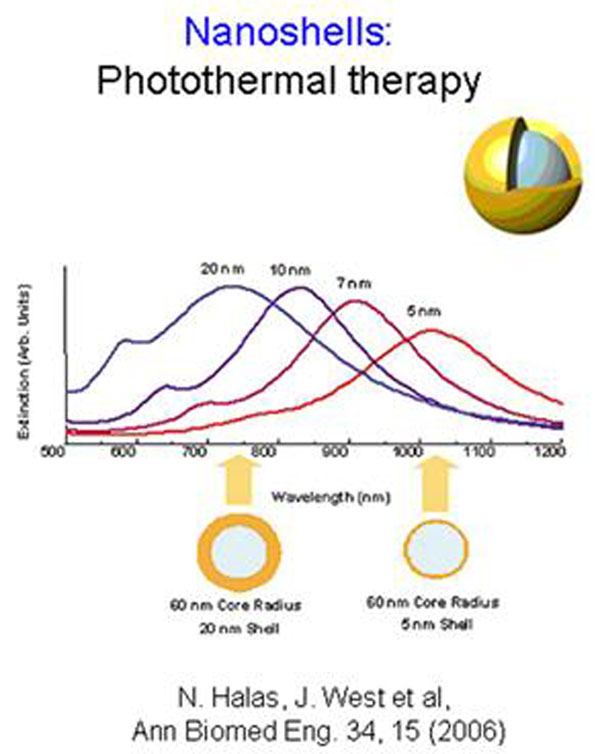
Dependencies of extinction, versus wavelength for nanoshells with different core radius

The paper presented could be considered as a recent review on application of nanoparticles and nanotechnology in cancer research. In the work [6], we could find basic research of American scientists in nanotechnology based for cancer research, (Figure 1).

## Conclusions

In conclusions we could say, that paper presented could be a successful tool for many medical scientists, physicians, molecular biology scientists, chemists etc. It’s give good knowledge, regarding nanotechnology based gold nanoparticles for cancer research. Also, some novel computational models, based on theoretical studies, analyzed here, could be developed in future inestigations.
